# Pentraxin 3 mediates neurogenesis and angiogenesis after cerebral ischaemia

**DOI:** 10.1186/s12974-014-0227-y

**Published:** 2015-01-24

**Authors:** Beatriz Rodriguez-Grande, Lidiya Varghese, Francisco Molina-Holgado, Olivera Rajkovic, Cecilia Garlanda, Adam Denes, Emmanuel Pinteaux

**Affiliations:** Faculty of Life Sciences, A.V. Hill Building, University of Manchester, Oxford Road, Manchester, M13 9PT UK; Health Sciences Research Centre, Roehampton University, Whitelands College, Holybourne Avenue, London, SW15 4SD UK; Department of Immunology and Inflammation, Humanitas Clinical and Research Center, Via Manzoni 56, Rozzano, MI 20089 Italy; Laboratory of Molecular Neuroendocrinology, Institute of Experimental Medicine, PO Box 67, Budapest, H-1450 Hungary

**Keywords:** Stroke, Inflammation, Pentraxin-3, Interleukin-1, Neurogenesis, Angiogenesis, Brain repair, Post-stroke recovery

## Abstract

**Background:**

The acute phase protein pentraxin 3 (PTX3) is a new biomarker of stroke severity and is a key regulator of oedema resolution and glial responses after cerebral ischaemia, emerging as a possible target for brain repair after stroke. Neurogenesis and angiogenesis are essential events in post-stroke recovery. Here, we investigated for the first time the role of PTX3 in neurogenesis and angiogenesis after stroke.

**Methods:**

PTX3 knockout (KO) or wild-type (WT) mice were subjected to experimental cerebral ischaemia (induced by middle cerebral artery occlusion (MCAo)). Poststroke neurogenesis was assessed by nestin, doublecortin (DCX) and bromodeoxyuridine (BrdU) immunostaining, whereas angiogenesis was assessed by BrdU, vascular endothelial growth factor receptor 2 (VEGFR2) and PECAM-1 immunostaining. *In vitro* neurogenesis and angiogenesis assays were carried out on neurospheres derived from WT or interleukin-1β (IL-1β) KO mice, and mouse endothelial cell line bEnd.5 respectively. Behavioural function was assessed in WT and PTX3 KO mice using open-field, motor and Y-maze tests.

**Results:**

Neurogenesis was significantly reduced in the dentate gyrus (DG) of the hippocampus of PTX3 KO mice, compared to WT mice, 6 days after MCAo. In addition, recombinant PTX3 was neurogenic *in vitro* when added to neurospheres, which was mediated by IL-1β. *In vivo* poststroke angiogenesis was significantly reduced in PTX3 KO mice compared to WT mice 14 days after MCAo, as revealed by reduced vascular density, less newly formed blood vessels and decreased expression of VEGFR2. *In vitro,* recombinant PTX3 induced marked endothelial cellular proliferation and promoted formation of tube-like structures of endothelial cell line bEnd.5. Finally, a lack of PTX3 potentiated motor deficits 14 days after MCAo.

**Conclusions:**

These results indicate that PTX3 mediates neurogenesis and angiogenesis and contributes to functional recovery after stroke, highlighting a key role of PTX3 as a mediator of brain repair and suggesting that PTX3 could be used as a new target for stroke therapy.

## Background

The acute phase protein pentraxin-3 (PTX3) is an important regulator of peripheral innate immunity and is now recognised as key mediator of inflammation during cardiovascular and cerebrovascular diseases [1,2]. Increased PTX3 levels are associated with the incidence of cardiovascular disease [3-5], and PTX3 is recognised as an independent predictor of mortality at 3 months after acute myocardial infarction [6]. Importantly, the involvement of PTX3 in cerebrovascular disease such as ischaemic stroke is emerging; peripheral PTX3 levels are strongly increased after experimental stroke in mice [1] whilst plasma PTX3 levels correlate with mortality after ischaemic stroke in human [7]. Importantly, our recent study found that PTX3 is highly expressed in the brain after experimental cerebral ischaemia and that its central expression is critically regulated by the cytokine interleukin-1 (IL-1), a pivotal regulator of inflammation after stroke. Whilst we found that PTX3 has no direct role in stroke pathogenesis, we found that PTX3 is a key regulator of early repair processes after stroke, including blood brain barrier (BBB) integrity, resolution of oedema and glial scar formation [1].

Inflammation is critically implicated in the pathogenesis of stroke and is regulated by IL-1 [8,9], whilst blocking IL-1-induced inflammation is neuroprotective in experimental and clinical settings [10,11]. Although acute IL-1-driven inflammation is detrimental to the brain and contributes to poor outcome, the delayed inflammatory response, although not well-documented, may initiate and sustain key brain repair mechanisms including neurogenesis and angiogenesis. For example, inhibition of matrix-metalloproteinase-9 (a key enzyme whose expression is driven by IL-1 [12]), impairs angiogenesis and functional recovery 7 to 14 days after stroke [13]. Angiogenesis takes place primarily in the penumbra surrounding the ischaemic core, correlates with survival after stroke [14] and is tightly linked with neurogenesis, since neural progenitor cells differentiate and migrate along angiogenic niches [15]. Although we have reported that IL-1-driven PTX3 expression in the brain is important for the early repair mechanisms associated with the vasculature, the role of PTX3 in delayed repair mechanisms, including neurogenesis and angiogenesis, has never been explored. The aim of this study was to characterize the role of PTX3 in post-stroke neurogenesis, angiogenesis and functional recovery.

## Methods

### Animals

PTX3 knockout (KO) mice, kindly provided by Dr. Cecilia Garlanda (Humanitas Clinical and Research Center, Rozzano, Italy), were bred as heterozygous, and litter genotyping was performed as described previously [16]. Age- and weight-matched wild-type (WT) and PTX3 KO male littermates were used for all experiments. IL-1β KO mice were provided by Prof. Yoichiro Iwakura (University of Tokyo, Japan). All mice were bred on a C57BL/6 background. Animals were kept at 21°C ± 1°C, 55% ± 10% humidity, in a 12 h light-dark cycle, and had free access to food and water. All animal procedures were carried out in accordance with the European Council directives (86/609/EEC) the Animal Scientific Procedures Act (UK) 1986 and the ARRIVE guidelines, and were approved by the Home Office (UK) (Project licence 40/3617).

### Cerebral ischemia induced by transient middle cerebral artery occlusion and BrdU administration

Transient middle cerebral artery occlusion (MCAo) was performed using the intraluminal filament method as described previously [17]. Briefly, a filament was introduced through the left external carotid artery and advanced along the internal carotid artery to occlude the middle cerebral artery (MCA). A drop in blood flow in the MCA territory was confirmed by laser Doppler monitoring (Moor Instruments, Axminster, UK). No difference in blood flow was observed between WT and PTX3 KO prior to, during and after occlusion [1]. After 30 min of occlusion, the filament was removed and mice recovered for 48 h, 6 days or 14 days. Mice were injected subcutaneously with 0.5 ml of sterile saline daily for rehydration, and were continuously monitored for neurologic symptoms. To assess cell proliferation, bromodeoxyuridine (BrdU, Sigma, Poole, UK) dissolved in sterile phosphate buffer saline (PBS) was injected intraperitoneally twice daily (50 mg/kg) in mice that recovered for 6 days in order to monitor the initiation of vascular proliferation as well as early neurogenesis. For mice recovering for 14 days, BrdU was injected from days 4 to 8 (once a day on days 4 and 8, and twice a day on days 5 to 7). This allowed us to study a later time window of proliferation still close to the peak of angiogenesis. BrdU injection during the first 3 days was avoided in this group to minimise BrdU signal from early glial proliferation, which is dominant at this time point.

### Tissue collection, measurement of infarct size and immunohistochemistry

Brains were collected and immunostained as described previously [1]. Briefly, mice were perfused transcardially with 0.9% saline followed by 4% paraformaldehyde (PFA). Brains were removed, fixed in 20% sucrose/PFA and sectioned (20- to 25-μm thickness) using a sledge microtome (Bright, Huntington, UK). Infarct size was determined as previously reported [18]. In brief, eight coronal sections per brain were stained with cresyl violet (Sigma, Poole, UK). Infarct area was measured on each section and corrected to account for oedema. Areas were then integrated to obtain infarct volume. For BrdU immunostaining, sections were pretreated with 1 M HCl for 2 min on ice, and then for 30 min at 37°C. For NeuN, doublecortin (DCX) and nestin immunohistochemistry, sections were pretreated for 30 min with 10 mM sodium citrate at 50°C. Nonspecific staining was blocked with 2% normal donkey serum (Jackson Laboratories, Baltimore, USA) in primary diluent (0.3% Triton X-100 in PBS) for 1 h. Overnight incubation in primary antibodies was followed by three washes and 2 h incubation in secondary fluorescent antibodies. Primary antibodies used were sheep anti-BrdU (1:500, Abcam, Cambridge, UK), rat anti-BrdU (1:500, AbD Serotec, Kidlington, UK), rat anti-PECAM-1 (1:200, BD Pharmingen, Oxford, UK), rabbit anti-VEGFR2 (1:300, Cell Signalling, Hitchin, UK), mouse anti-NeuN (1:500, Millipore, Watford, UK), mouse anti-nestin (1:500, Millipore, Watford, UK) and goat anti-DCX (1:750, Santa Cruz Biotechnologies, Dallas, Texas, USA). Secondary antibodies were conjugated to Alexa 488 or 594 fluorochromes (Invitrogen, Paisley, UK). Sections were cover-slipped with Prolong Gold antifade with or without DAPI (Invitrogen, Paisley, UK). An Olympus BX51 upright microscope coupled to a Coolsnap ES camera (Photometrics, Herts UK) and MetaVue Software (Molecular Devices, Sunnyvale, California, USA) were used to capture the images. Two coronal sections per animal were analysed with three randomly selected images within each area of interest (striatum, SVZ or hippocampus, depending on the marker assessed). Coronal sections from Bregma levels 1.18 to 0 (according to the MBL mouse brain atlas, www.mbl.org) were used to quantify markers in the striatum and the SVZ, and sections from Bregma levels −1.46 to −2.54 were used to quantify markers in the hippocampus.

### Image analysis

Counts of BrdU positive nuclei, BrdU-PECAM-1 positive vessels and nestin positive profiles were done manually. Areas and mean optical density (OD) were measured using Image J (National Institutes of Health, USA, http://imagej.nih.gov/ij/). The percentage of the total area of the image stained with PECAM-1 or VEGF was quantified. Percentage of area stained was measured manually after thresholding the images with Image J. PECAM-1-BrdU and VEGF measurements are presented as raw data and also normalised to the amount of PECAM-1 (for example, (%VEGF staining/%PECAM-1 staining) x 100). In all cases, the average of the values obtained from the six images (three images in each of the two sections used) was calculated.

### Behavioural tests

Mice recovering for 14 days were tested for behavioural deficits. Basic motor deficits were measured with a points system adapted from a previous study [19] as follows: 1 point for torso flexion to the right in the air; 2 points for deficit in gripping with a paw; 3 points for circling with the front paws when suspended from the tail; 4 points for spontaneous circling on the floor; 5 points for irresponsiveness to stimuli. The sum of all points was used as the score of motor deficit. General motivation to explore and motor function were measured in an open field: mouse movement within a square chamber was tracked for 5 min and analysed by overlapping the recording to a 16-squares grid with 2020 PLUS tracking software (HVS Image, Thornborough, UK). Time mobile, number of line crossings, number of rearings and rotational bias (asymmetric rotation that occurs as a consequence of unilateral MCAo) were measured. Short-term memory was measured in a Y-maze as described previously [20]. Briefly, mouse entries in each arm of a Y-shaped chamber containing a different visual stimulus at the end of each arm were measured during 8 min. The percentage of alternations (that is ABC, CBA, BAC, *etcetera*) was calculated. Y-maze test was performed on the evening of day 13 after MCAo, and assessment of motor scores and open field test were performed on the morning of day 14 after MCAo, when mice were euthanized.

### Neural stem progenitor cell cultures and measurement of neurogenesis *in vitro*

Neural stem progenitor cell (NSPC) cultures were prepared from the cerebral cortex of WT or IL-1β KO mice (embryonic stage 16 days of gestation) as previously described [21]. Cells were then cultured as free-floating neurospheres in 75 cm^2^ flasks (Nunc, Roskilde, Denmark) at a density of 20,000 cells/ml [22] in DMEM/F12 supplemented with B27, 3 μg/ml heparin, 20 ng/ml fibroblast growth factor (FGF) and 20 ng/ml epidermal growth factor (EGF) (PeproTech, London, UK). Primary neurospheres were grown for 7 to 10 days before secondary cultures were established with mechanically dissociated cells. Neurospheres were passaged every 5 to 7 days, and experiments were performed after three to seven passages.

NSPC proliferation was assessed using a well-accepted measure of neurosphere proliferation [23,24]. NSPCs were progressively diluted from 4,000 to 125 cells in 96-well plates. Dissociated cells were exposed for 7 days to a single optimal concentration of PTX3 (100 ng/ml; R&D Systems, Abingdon, UK), after which the number of neurospheres formed was counted. The number of new neurospheres was plotted against the initial number of cells plated, from which the fate of the neural stem cells was evaluated [25,26]. As an additional measure of the effect of PTX3 on *in vitro* neurogenesis, BrdU incorporation into neurospheres was quantified. After 6 days in culture, cells were treated with PTX3 (100 ng/ml) for 24 h. BrdU (10 μM) was added in the last 18 h. Neurospheres were then dissociated, plated onto poly-L-ornithine coated (15 μg/ml) 24-well plates for 1 h, fixed for 20 min in 4% PFA and immunostained. For BrdU immunostaining, cells were treated with 2 M HCl for 10 min, which was neutralized with 0.1 M borate buffer, pH 9.5. BrdU was visualized with a BrdU monoclonal antibody (clone MoBU-1) Alexa Fluor conjugate 594 (Invitrogen, Paisley, UK). BrdU incorporation was defined by the cells labelled by Hoechst 33342 (10 μM) and with the BrdU antibody. For *in vitro* neurogenesis assays, images were acquired using a fluorescence microscope (Olympus BX51), and neurospheres were counted using the image analysis software ViewFinder 3.0.1 (Pixera Corporation, Santa Clara, California, USA). Six microscope fields were counted in each well at a 10× magnification, and two to four wells were used per treatment in each experiment.

### Measurement of angiogenesis *in vitro*

To assess the effect of PTX3 on angiogenesis *in vitro*, a mouse endothelial cell line bEnd.5, which closely resembles primary brain endothelium in culture, was used as previously reported [27]. Since we used a model of transient ischaemia in which blood flow is restored after the surgery, we decided to carry out our *in vitro* experiments in normal conditions of oxygen and glucose such as those present in the brain at the time when angiogenesis and neurogenesis occur *in vivo*. Cells were grown in DMEM (high glucose, 4.5 g/l; Invitrogen, Paisley, UK), supplemented with 10% FCS, 1% nonessential amino acid, 2 mM glutamine, 1 U/ml penicillin, and 100 mg/ml streptomycin. To assess the proliferative effect of PTX3, cells were seeded at 50,000 cells/cm^2^ and were subsequently treated with recombinant PTX3 at a concentration of 100 or 1,000 ng/ml. After 48 h, cellular proliferation was observed under bright field microscope, and cells were counted on five random fields taken from three separate cultures.

### Statistical analysis

Data normality was tested using Shapiro-Wilk test, and normally distributed data were analysed as follows. An unpaired Student’s t-test was used to compare two groups. For the comparison of more than two groups, a one-way or two-way ANOVA followed by Bonferroni’s post-hoc test was used. For the comparison of means to a given value, one sample t-test was used. For motor scores, which were not normally distributed, the non-parametric Mann-Whitney test was used. All graphs show mean ± SEM, except for motor deficit scores, in which medians and interquartile ranges have been used. Statistical significance was set at *P* <0.05. All analyses were made using GraphPad Prism 5.0 (GraphPad Software Inc., USA).

## Results

### PTX3 regulates neurogenesis after middle cerebral artery occlusion

**Figure 1:**
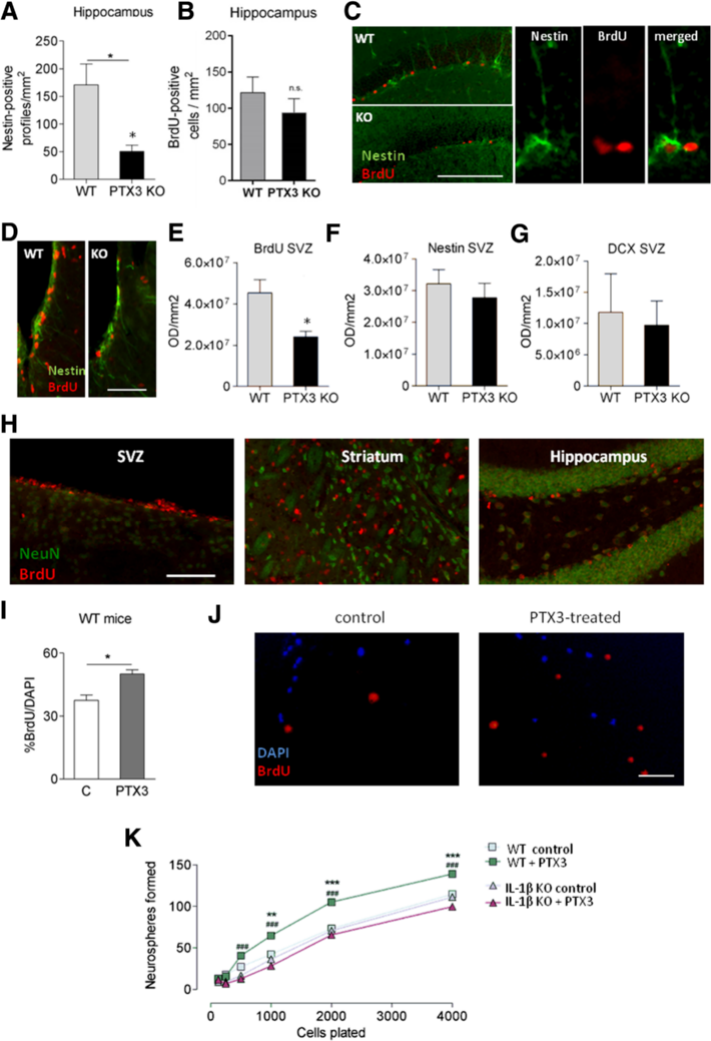
**PTX3 promotes neurogenesis** ***in vivo*** **after middle cerebral artery occlusion** **(MCAo) and** ***in vitro*** **.** Less nestin-positive profiles are found in the dentate gyrus (DG) of PTX3 knockout (KO) mice compared to wild-type (WT) mice 6 days after MCAo **(A)** although BrdU levels in that area are not significantly different **(B)**. BrdU-nestin double immunofluorescence reveals some proliferating nestin-positive cells **(C)**. BrdU-nestin staining in the subventricular zone (SVZ) **(D)** shows that PTX3 KO mice have significantly less BrdU staining than WT mice in that area 6 days after MCAo **(E)**, and the same trend, although not significant, is observed for nestin **(F)** and DCX **(G)**. Images show no colocalisation between BrdU and NeuN in the SVZ, striatum or hippocampus 14 days after MCAo, indicating that newly formed neurones might not be mature at this time point **(H)**. PTX3 (100 ng/ml) induces neural stem progenitor cell (NSPC) proliferation, as shown by BrdU/DAPI ratio after 24 h treatment **(I, J)**. BrdU and DAPI staining in NSPC cultures **(J)**. PTX3 (100 ng/ml) treatment of NSPCs for one week increases neurosphere formation in WT, but not in IL-1β KO cultures **(K)**. Scale bars: 200 μm **(A)**, 80 μm **(D)**, 100 μm **(H)**, 50 μm **(I)**. Students t-test (**(A)**, n = 4 to 6; **(B)**, n = 4 to 5; **(E)**, **(F)** and **(G)**, n = 5; **(I)**, n = 4) or two-way ANOVA followed by Bonferroni’s post-hoc test (**(K)**, n = 4) were used. *P <0.05, **P <0.01, ***P <0.001, ###P <0.001. In **(K)**,*WT control versus other experimental groups, #WT PTX3 versus IL-1β KO PTX3. Error bars show SEM.

Our previous study found no significant difference in infarct size between PTX3 KO and WT mice 48 h or 6 days [1] after MCAo. We found, however, that PTX3 is a key regulator of neurogenesis after experimental cerebral ischaemia; nestin-positive NSPCs in the dentate gyrus (DG) of PTX3 KO mice were less abundant than in WT mice (Figure 1A) 6 days after MCAo. Some of these nestin-positive NSPCs were proliferating cells, as shown by BrdU immunostaining (Figure 1C). There was no significant difference between genotypes in the levels of BrdU staining in the hippocampus (Figure 1B), but PTX3 KO mice had significantly less BrdU staining in the subventricular zone (SVZ) (Figure 1D and E), and a similar trend, although not significant, was observed for nestin (Figure 1D and F) and DCX staining (Figure 1G). No BrdU, nestin or DCX-positive cells were seen in the contralateral hemisphere (except for a low number of BrdU-positive cells in the SVZ where proliferation of progenitor cells is seen even at baseline [28] (data not shown)). However, newly formed mature neurones (BrdU-NeuN positive) were not found in any area of the brain, not even 14 days after MCAo (Figure 1H).

To confirm our *in vivo* observations, we tested the effect of PTX3 on neurogenesis *in vitro*; treatment of WT neurosphere cultures with recombinant mouse PTX3 resulted in an increase in proliferation indicated by an increase in BrdU incorporation (Figure 1I and J). The number of neurospheres was also significantly increased after PTX3 treatment of WT cells, and this effect was absent in IL-1β KO cultures (Figure 1K), suggesting that endogenous IL-1 is necessary for the neurogenic effect of PTX3.

### Lack of PTX3 impairs angiogenesis after middle cerebral artery occlusion

**Figure 2:**
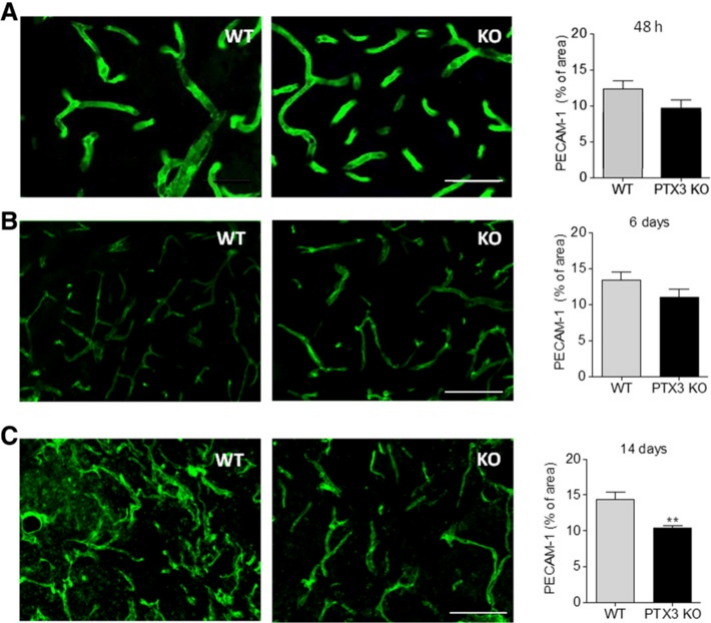
**Lack of PTX3 reduces striatal vascular density during the recovery phase after middle cerebral artery occlusion** **(MCAo).** PECAM-1 staining in the striatum of wild-type (WT) and PTX3 knockout (KO) mice was quantified, showing that PTX3 KO mice have significantly less vasculature 14 days after MCAo **(C)**. A similar trend, although not significant, is seen 48 h **(A)** or 6 days **(B)** post MCAo. Scale bars = 50 μm. Students t-test (n = 4) was used. **P <0.01. Error bars show SEM.

**Figure 3:**
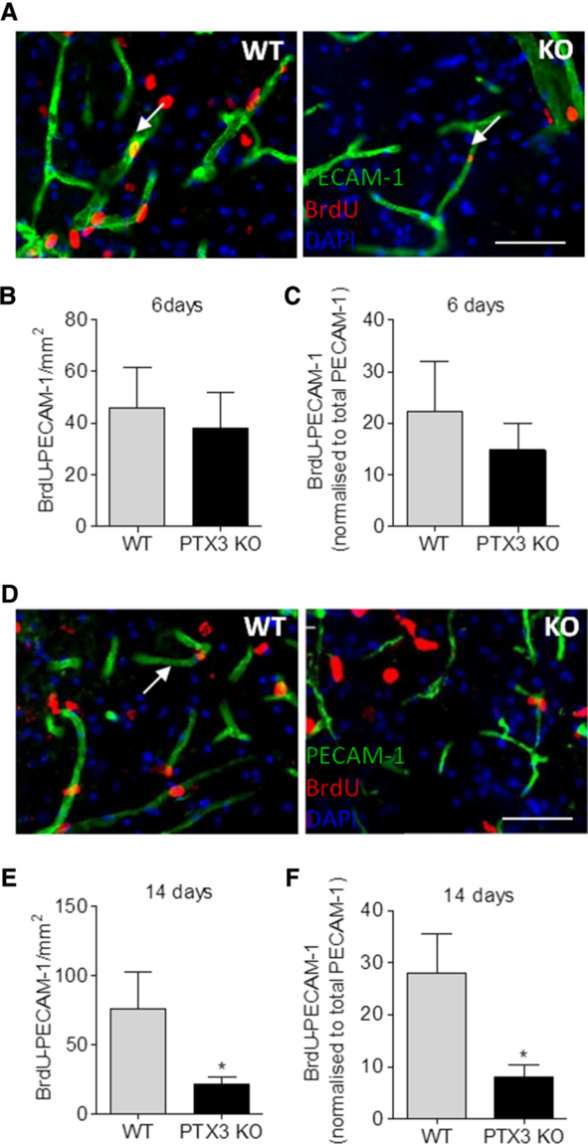
**Lack of PTX3 impairs angiogenesis 14 days after middle cerebral artery occlusion** **(MCAo).** Proliferating vessels (indicated by white arrows in **(A)** and **(D)**) are seen in the striatum 6 days **(A)** and 14 days **(D)** after MCAo. Angiogenesis is similar for both genotypes 6 days after MCAo **(B)**, but PTX3 knockout (KO) have less striatal proliferating vessels than wild-type (WT) mice 14 days after MCAo **(E)**, even after normalisation with the total amount of PECAM-1 **(E)** and **(F)**. Scale bars = 50 μm. Students t-test (n = 4) was used. *P <0.05. Error bars show SEM.

**Figure 4:**
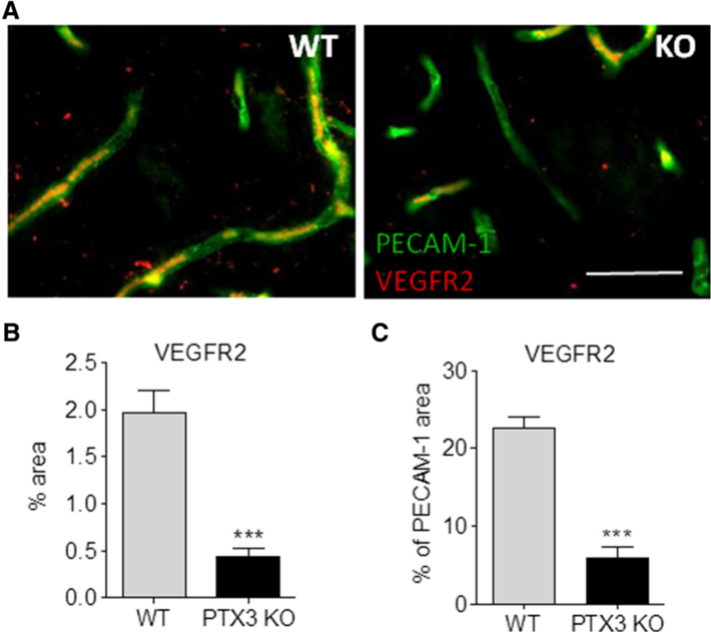
**VEGFR2 is reduced in PTX3 knockout (KO) mice 14 days after middle cerebral artery occlusion (MCAo).** Quantification of VEGFR2 immunostaining (red in **(A)**) indicate that PTX3 KO mice have a significantly reduced amount of VEGFR2 **(B)**, even when normalised with the levels of PECAM-1 (green) **(C)**. Scale bars = 50 μm. Students t-test (n = 4) was used. ***P <0.001. Error bars show SEM.

Since neurogenesis is coupled with angiogenesis in the injured brain [15], we tested whether PTX3 also promotes angiogenesis after experimental stroke. Lack of PTX3 did not significantly affect the levels of PECAM-1 staining 48 h (Figure 2A) or 6 days (Figure 2B) after MCAo, although there was a trend towards decreased PECAM-1 staining in PTX3 KO mice. In contrast, PECAM-1 staining was significantly reduced in PTX3 KO mice 14 days after MCAo, compared to WT mice (Figure 2C). To confirm that this variation in PECAM-1 staining was due to angiogenesis, we quantified the amount of BrdU incorporated within the vascular endothelium. After 6 days (Figure 3A,B and C) and 14 days (Figure 3D,E and F) post-MCAo, several blood vessels had incorporated BrdU. Lack of PTX3 significantly affected the amount of proliferating vessels 14 days (Figure 3E) but not 6 days (Figure 3B) after MCAo. To determine if the reduced amount of vascular BrdU was only due to the fact that PTX3 KO mice had fewer blood vessels, we normalised the amount of BrdU to the amount of PECAM-1 staining (Figures 3C and F). This also indicated a significant deficit in angiogenesis in PTX3 KO mice 14 days after MCAo. In addition to fewer proliferating vessels, PTX3 KO mice had less vascular VEGFR2 staining 14 days after MCAo (Figure 4A), both as percentage of the total area (Figure 4B) and when normalised and expressed as percentage of the PECAM-1-positive area (Figure 4C). VEGF exerts a potent pro-angiogenic effect through the activation of VEGFR2 [29], whose levels increase after ischaemia [30].

**Figure 5:**
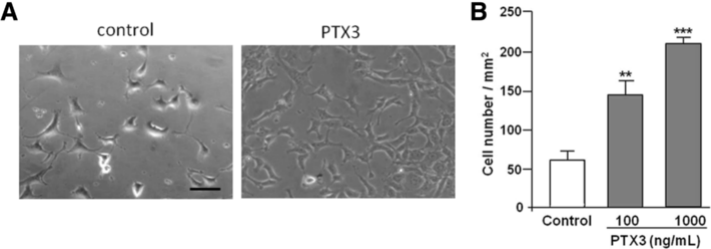
**PTX3 increases proliferation of endothelial cells** ***in vitro*** **. (A)** Mouse brain endothelial cells (bEND5) treated with PTX3 for 48 h (100 ng/ml) proliferate more than controls (phosphate buffered saline (PBS)-treated). **(B)** There is a significant and dose-dependent increase in the number of bEND5 cells treated with PTX3. Scale bar: 50 μM. **P < 0.01, ***P <0.001, using One-way ANOVA with Dunnett’s post-hoc test (n = 4). Error bars show SEM.

We also tested the effect of PTX3 on cellular proliferation *in vitro*, using a mouse endothelial cell line, bEnd.5. The addition of PTX3 to bEnd.5 cultures induced a significant increase in cellular proliferation (Figure 5A,B). Morphological assessment of cultures showed that cells formed tube-like structures confirming the potent pro-angiogenic effect of PTX3.

### Lack of PTX3 leads to worse functional recovery after experimental stroke

**Figure 6:**
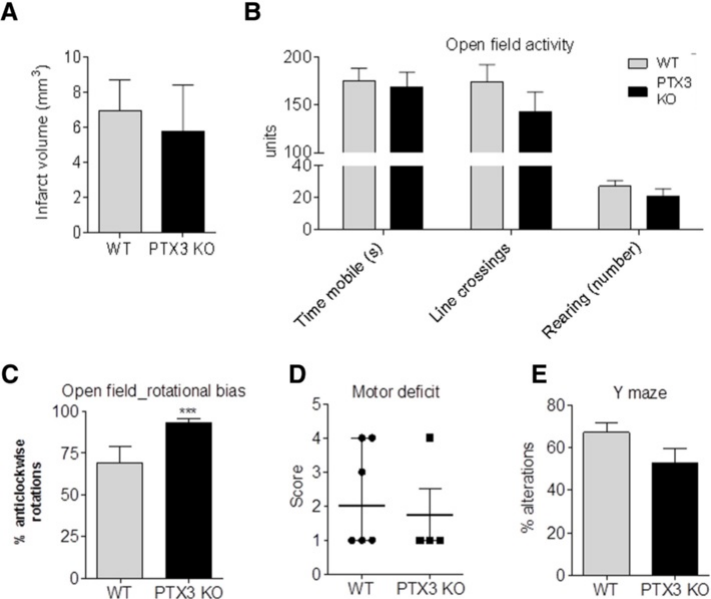
**PTX3 knockout (KO) mice have increased rotational bias 14 days after middle cerebral artery occlusion (MCAo).** No significant difference is observed in infarct size between genotypes 14 days after MCAo, as quantified on cresyl violet-stained coronal brain sections **(A)**. Open field test revealed no difference in parameters reflecting general anxiety and activity such as time mobile, number of line crossings and number of rearings **(B)**. Rotational bias was significant in PTX3 KO but not in wild-type (WT) mice **(C)** and general motor activity was not affected by the lack of PTX3 **(D)**. Short term memory, measured as the percentage of alternations made on a Y-maze, was not affected **(E)**. Student´s t-test (**(A)**, **(B)**, **(C)** and **(E)**, n = 4 to 6), one-sample t-test versus 50% (**(C)**, n = 4 to 6) and Mann-Whitney tests (**(D)**, n = 4 to 6) were used. ***P <0.05 were performed. In C, *** corresponds to the result of the one-sample t-test versus 50%. Error bars show SEM (**(A)**, **(B)**, **(C)** and **(E)**) or interquartile range **(D)**.

Increased neurogenesis has been linked with improved neurobehavioral function after stroke [31]. To determine whether the lack of PTX3 had an effect on behavioural outcome, several tests were carried out analyzing basic parameters of activity, exploratory behaviour, motor function and memory, 14 days after MCAo, when no difference in infarct size between genotypes was observed (Figure 6A). The amount of time mobile, the number of lines crossed and the number of rearings within an open field test were used to study alterations in general activity and exploratory behaviour, but no significant differences between genotypes were identified (Figure 6B). The proportion of anti-clockwise rotations was significantly higher in PTX3 KO mice (93% anti-clockwise rotations on average, compared to the 50% that would correspond to a healthy mouse) (Figure 6C). Although not significant, WT mice also displayed rotational bias (Figure 6C). Rotational bias indicates a lateral motor deficit, consequence of the unilateral MCAo. The score reflecting general motor activity, however, did not vary between genotypes (Figure 6D). Short term memory, measured in a Y maze, was not significantly affected by the lack of PTX3, although a trend indicated that PTX3 KO mice could have a slight memory deficit compared to WT mice (Figure 6E).

## Discussion

Here we show for the first time that the acute phase protein PTX3, a biomarker of stroke severity [7], is a key regulator of neurogenesis and angiogenesis after cerebral ischaemia, and that it has an impact on the recovery of motor function.

The role of PTX3 in neurogenesis had never been studied. PTX3 has been shown to participate in other post stroke responses such as resolution of oedema and glial scar formation [1]; here we observe that, 6 days after the ischaemic event, PTX3 promotes repair in the form of neurogenesis, especially in the hippocampus. However, since nestin only marks an early stage of differentiation [32], further research is required to explore whether PTX3 has an effect in the final stages of NSPC differentiation and functional integration. We observed a decrease in BrdU staining, but not in NSPCs in PTX3 KO mice in the SVZ. This could be due to a decrease in glial proliferation in PTX3 KO mice which has been reported after stroke [1]. It is worth mentioning that infarct size did not significantly differ between WT and PTX3 KO mice at this time (6 days post-MCAo) [1]. Although there was a trend towards a bigger infarct size in PTX3 KO mice at that time, the opposite trend was observed 14 days after MCAo, suggesting that the lack of PTX3 is not a strong modulator of infarct size. Other parameters affected by the lack of PTX3 such as BBB breakdown [1] could indirectly affect early neurogenesis. However, our *in vitro* findings, which show that PTX3 treatment induces NSPC proliferation, indicate that PTX3 is a direct endogenous neurogenic mediator. Interestingly, the neurogenic effect of PTX3 *in vitro* was abrogated in IL-1β KO cultures. IL-1 is known to facilitate neurogenesis [33], and our results suggest that PTX3 could be involved in that process.

The role of PTX3 in angiogenesis is more controversial. In peripheral tissues, PTX3 has been described as an anti-angiogenic factor *in vitro* and in matrigel or alginate bead implants *ex vivo* [34,35]. In contrast, our data show that PTX3 exerts pro-angiogenic actions on the cerebrovascular endothelium, indicating that PTX3 actions could be different in the brain after injury compared to peripheral organs. Indeed, our earlier studies showed that central and peripheral PTX3 expression is differently regulated [1]. Supporting a beneficial role for PTX3 in angiogenesis, the lack of PTX3 reduced the amount of capillaries in the reperfused area, as well as inducing a worse outcome in a study of cardiac ischaemia [36]. The effect of PTX3 in other mechanisms of repair [1] may benefit the overall recovery and indirectly facilitate angiogenesis, overruling the anti-angiogenic actions of PTX3. Baldini and colleagues found elevated levels of both PTX3 and VEGF in samples of patients suffering from arterial inflammation [37], and we observed a lower amount of VEGFR2 in blood vessels of PTX3 KO mice. VEGF is thought to be produced by astrocytes after cerebral ischaemia [38,39] and then binds to VEGFR2 in endothelial cells, promoting angiogenesis [40]. Lower VEGFR2 levels could reflect a lower level of VEGF. Further research is required to explore the role of PTX3 in VEGF production. Since microglial-derived factors can also promote angiogenesis [41], reduced microglial proliferation (which is also observed in PTX3 KO mice [1]) could be an additional mechanism by which the lack of PTX3 impairs angiogenesis. Decreased angiogenesis in PTX3 KO mice could partly account for a decreased amount of neurogenesis since it is well known that both processes are linked [15]. Interestingly, IL-1 promotes angiogenesis [42] as well as neurogenesis [33]. After cerebral ischaemia, IL-1 is known to drive PTX3 expression [1], which would, in turn, facilitate angiogenesis and neurogenesis. These observations highlight the importance of PTX3 and IL-1 in brain repair. Discriminating between early IL-1 noxious actions [11] and late IL-1 repair-promoting actions, such as those observed here, is necessary to choose the appropriate time of administration of anti-inflammatory treatments. This is essential to avoid unwanted blockade of repair mediators such as PTX3, which require a certain degree of pro-inflammatory molecules.

In terms of behaviour, lack of PTX3 caused a significant increase in rotational bias, which occurs as a consequence of the unilateral brain damage and reflects asymmetric motor function [43] and may indicate the slower recovery of contralateral motor function in PTX3 KO mice. Since there were no new mature neurones (NeuN-positive) at the time when behavioural tests were performed, differences in behaviour might not be attributed to differences in neurogenesis alone. Reduced angiogenic responses in PTX3 KO are likely to contribute to worse tissue perfusion and delayed reorganization of injured neuronal pathways. Reduced angiogenesis could also affect the function of surviving neurones, and reduced angiogenic responses could contribute to delayed recovery after stroke [44].

## Conclusions

We have shown here that PTX3 is a key mediator of angiogenesis and neurogenesis after cerebral ischaemia, and has a beneficial impact on recovery of lateral motor function. These observations highlight further the relevance of PTX3 as a potential target for stroke recovery. In the long term, this could have important clinical implications given the increasing incidence of stroke and the lack of treatments targeting the recovery phase.

## Abbreviations

BBB: blood–brain barrier
BrdU: bromodeoxyuridine
DCX: doublecortin
DG: dentate gyrus
EGF: endothelial growth factor
FGF: fibroblast growth factor
IL-1: interleukin-1
KO: knockout
MCA: middle cerebral artery
MCAo: middle cerebral artery occlusion
NSPC: neural stem cell progenitor cells
OD: optical density
PBS: phosphate buffered saline
PFA: paraformaldehyde
PTX3: pentraxin 3
SVZ: subventricular zone
VEGF: vascular endothelial growth factor
VEGFR: vascular endothelial growth factor receptor
WT: wild type
